# Early Operation in Appendicitis, with a Case

**Published:** 1894-12

**Authors:** J. McFadden Gaston

**Affiliations:** Atlanta, Ga., Professor of Principles and Practice of Surgery, Southern Medical College, etc.


### EARLY OPERATION IN APPENDICITIS, WITH A CASE.*

BY J. McFADDEN GASTON, M.D., ATLANTA, GA.,

Professor of Principles and Practice of Surgery, Southern Medical College, etc.

Upon making some remarks on the paper of Dr. J. B. Murphy before the surgical section of the American Medical Association at Milwaukee in 1893, I was asked by Dr. Nicolas Senn, if I would recommend an operation in all cases of appendicitis. My reply was, that whenever the diagnosis was clearly established, I considered the operation warranted, as delay only increased the danger likely to attend perforation of the appendix.

It is evident from the reports of cases supposed to be appendicitis having a favorable termination with expectant treatment, that operative measures are not requisite in all inflammatory processes involving the iliac region. But we must admit that the former classification of typhlitis, paratyphlitis and perityphlitis was not entirely without foundation in assigning their troubles to the cecum and surrounding structures. This is emphasized by the facts developed in not a few instances of operations for supposed appendicitis, when it is found that the case is an iliac abscess without any involvement of the appendix vermiformis. A striking illustration of this doubtful diagnosis is reported in the *Atlanta Evening Journal* of November 5th, I presume without professional authorization, in which it is stated that the patient had been sick about a week with a trouble that looked like appendicitis, but he recovered and was able to be out upon the streets. After being up for three days his fever returned and his physician, after consulting with two other colleagues, decided that there was an abscess in or near the appendix which would prove fatal if it was ruptured. The trouble was near the appendix and the patient submitted to the operation, but it is stated that he is now grieving because the doctors failed to remove it.

---

*Read before the Southern Surgical and Gynecological Association, at Charleston, S. C., November 13, 1894.

The inference is that it was not involved, and hence it was not excised; although the patient had said to the doctor, "I want you to cut off that appendix whether there is anything the matter with it or not," so as to be free from future trouble.

This result is in keeping with the statements published through medical channels, that it is difficult to decide in advance of an operation whether the origin of these inflammations in the ileo-cecal region is located in the appendix or adjacent structures.

This brings us back then to my position that whenever the diagnosis is clearly established, the operation is warranted, and, I may say, is demanded, without delay.

But the fundamental difficulty of making out the precise location of the trouble and diagnosticating unmistakable appendicitis at an early stage of its development, confronts the surgeon at the outset and must be resolved before proceeding to perform any radical surgical operation.

After the pelvic inflammation has gone on until there is purulent collection which can be detected by palpation, the case no longer requires any acumen for diagnosis or skill in the procedure for its relief. It is nothing more than a circumscribed abscess, which plainly calls for evacution by an incision and antiseptic dressings. The danger point has been passed under the régime of masterly inactivity in a case of this nature, and it only needs the application of the simplest principles of minor surgery to carry it to a successful termination.

But the great importance of recognizing the incipient stage of appendicitis lies in the risk of perforation, with all its appalling consequences, before there are any patent signs of surrounding disturbance, and here comes the life-saving surgery of appendicitis.

He who can detect a diseased appendix which threatens perforation or discern the fact of its perforation immediately after its occurrence, and takes time by the forelock with prompt surgical measures, is the great deliverer.

It has fallen to my lot to observe the serious consequences of perforation of the appendix vermiformis in several cases, and the *post mortem* examinations of these fatal results have been reported from time to time during the past twelve years. Some of these cases could have been rescued undoubtedly by a timely operation, and they have served as an incentive for urging a resort to early operative measures so soon as the nature of the disease could be known by the physical signs accompanying appendicitis.

In a paper read before the American Medical Association in the spring of 1886, the following language was quoted by me from Ziegler: *

"The vermiform appendage is peculiarly adapted to catch and retain substances passing through the cecum. Matters which have been swallowed—such as grape-seeds, apple-pips, cherry-stones and the like—and feces, may accumulate in the appendage and set up inflammation. Sometimes these become crusted with phosphates and carbonates, and so form fecal concretions or calculi. The inflammation thus set up may extend to all the coats of the appendage, and then attack the contiguous structures, and in this way necrosis and gangrene with perforation may be caused."

The effects produced by perforation of the vermiform appendix vary with the anatomical relations of the parts and the seat of the ulceration. In illustration of this point, I quoted in my paper laid before the American Medical Association in June, 1887, the high authority of Mr. F. Treves, as follows: "The appendix commonly lies behind the end of the ileum and its mesentery, and is directed upwards and towards the left. In the only other common position, it ascends vertically behind the cecum. It may, however, be so placed that its free end lies at the brim of the pelvis. If perforation takes place near the attached end, or if the whole tube lies behind the cecum, the abscess would be in the same situation as that resulting from disease of the cecum, and would be indistinguishable from it. When the appendix occupies its more common situation, and when the perforation occurs in the free part, it is followed either by general peritonitis, usually fatal under a week, or by the formation of a collection of pus inclosed in a cavity formed by the surrounding coils of intestines firmly united to each other by adhesions."

According to Dr. Fenwick, in ninety-five cases of which accurate details could be obtained, thirty-eight presented localized collections

*Text-Book of Pathological Anatomy and Pathogenesis. Art. 471, page 638.

of pus. It is very unfavorable when perforation takes place before adequate adhesions are formed. Fatal peritonitis is nearly always induced.

I went on to state that in cases of doubtful diagnosis, presenting reasonable grounds for the conviction that there exists a disorder calling for operative interference, exploratory laparotomy should be resorted to at an early stage after other modes of treatment have failed to afford relief. The consequences of thus opening the abdominal cavity when operations upon the contained organs are not undertaken, are not generally serious. Operations in the ileo-cecal derangements should not be delayed until the physical powers have become prostrated, but resorted to while there is capacity for reaction of the vital forces.

In another paper, read before the same body at the meeting in May, 1888, I remarked that while some have claimed that inflammation is set up in the vermiform appendage as a result of derangement of the digestive apparatus, and others have supposed that the entrance of germs into its cavity has developed morbid processes in its structure, the facts go to prove that such causes of disease are rare in comparison with the troubles growing out of local irritation having a mechanical origin.

There is absence of preliminary indications pointing to the nature of the inflammatory process at the incipiency of disorders of the appendix vermiformis, which presents a problem of difficult solution for the surgeon. At this early stage, however, there may exist a certain degree of sensitiveness upon pressure, immediately over the site of the appendix, and this tenderness, revealed by cautious palpation, may serve for the recognition of an incipient inflammation, when there is no perceptible thickening or induration in the tissues of the appendix. It is clear that the causative influence, of whatever character it may be, must, up to a certain point, be of gradual operation in the development of inflammation. During this stage, it is of the greatest importance to be able to recognize its presence and progress. Not only the external examination should be carefully made, but a digital exploration by the rectum should be directed to the discovery of the seat of the inflammation in the appendix.

There are in most instances some concomitants of the local trouble, manifested in derangement of the alimentary canal, perhaps of a reflex order, prior to the extension of the inflammatory process to the adjoining structures. In the early history of appendicitis, it has been noted that there may occur, either a disposition to frequent evacuations, or a state of intestinal torpor, in which it is difficult to arouse the peristaltic action of the bowels, either by purgative medicines or an enema. During this period the constitutional disturbance is not very marked, and yet there exists usually a febrile state, with slight rises of temperature, so that nice discrimination will detect a departure from the normal condition of the system.

It is found generally that inflammation set up in the wall of the canal by a mechanical irritant leads to perforation and extravasation of the contents, accompanied by characteristic shock and followed by diffused inflammation. This is the dividing line between two distinct phases of symptoms, and whatever may have obscured the case previously, it is for the most part well defined after this grave result.

There may be only a circumscribed involvement of the surrounding structures, exciting adhesive inflammation in the serous tissues which shuts in the exudation from the cavity of the appendix, and thus forms a local abscess. But usually the septic matter permeates in different directions and sets up general peritonitis, with a train of constitutional troubles. With the uncertainty that hangs over the diagnosis in the early stages of appendicular disorders, the expectant treatment is generally recognized as the most prudent course, but there is a growing tendency among surgeons to cut the Gordian knot by a decisive step, and resort to an exploratory operation by incision for determining the exact seat and character of the disturbance. Some aggressive practitioners have inculcated surgical interference at the earliest practicable period, and with the lights before us in cases of this kind, the surgeon is warranted, so soon as he has good and sufficient reasons to believe that there exists progressive inflammation of the appendix, in verifying this diagnosis by cutting down upon it. This course is indicated when the symptoms of a local and constitutional nature are such as to raise a presumption in favor of appendicular inflammation, in advance of those phenomena which usually accompany perforation. In other words, an exploratory operation is advisable, with a view to learn the exact condition of the parts involved, and in the event there is no evident manifestation of inflammatory action in the tissues of the appendix or cecum, and no indurated mass can be discovered in either by careful exploration, the incision may be closed without any probability of serious consequences.

If, however, on the contrary, appendicitis is verified, with or without the recognition of a solid body within its canal, it would be proper to ligate and excise the appendix vermiformis as a security against further inflammatory developments in its structure. Thus all liability to the graver results of perforation would be most effectually obviated, and thus surgery of a destructive order would eventually prove conservative in preventing other grave consequences. The diagnosis of appendicitis clearly cannot be guaranteed without an exploratory operation, as the statements of those most familiar with this class of cases show most conclusively. It does not augment materially the risk to life, while it insures a radical cure when disease exists.

I would not be understood to favor a resort to the trocar or other mode of puncture to reach a supposed purulent collection in the iliac region, as it is evident that no practical utility results from such procedure, and I am convinced that great harm may ensue from such explorations.

I am on record for the statement, in 1888, that "if there be a doubt in the diagnosis of appendicitis, it is solved by a transverse incision in the iliac region, and if nothing be revealed requiring further surgical interference, the opening may be closed by suturing the peritoneum with catgut in the continuous form of suture, while the fascia, muscles, and skin are brought together by interrupted suture of iron-dyed silk. There will not occur much strain upon the suture in this line of union, and the operation should not be carried further, except when the evidence of disease in the adjacent structures demands a larger field of observation." If this is done, with the precautions suggested recently by Dr. Chas. McBurney for separating the muscles without cutting their fibers, it will still further simplify this procedure.

In a case of appendicitis not involving any doubt in the diagnosis, I have concluded that an incision of three or four inches made outside of the right rectus muscle, extending perpendicularly downward over the cecum, will afford the best line of access to the appendix. In case a suppurating tract should be discovered extending upwards towards the liver, the opening may be carried up along the linea semilunaris to any extent which may be requisite for exposing the sulcus or the sinus containing pus. Observation of the difficulties encountered in exploring the right iliac region by an incision of the linea alba has led to its abandonment.

This brief outline of the incipiency, progress, and termination of appendicitis which was presented by me at an early period of the history of this disease, holds good to-day. While others have added to the practical details of treatment during the past six or eight years, I realize that a more precise and definite description of appendicitis has not been given than that afforded in my papers. They were laid before the profession in the years 1886, 1887, and 1888, and will be found published in the "Journal of the American Medical Association" for those years, to which members are referred.

Having observed in some cases of appendicitis a peculiar pain in the penis and noticing that others have drawn attention to the same complication, it is not out of place to record that this feature was very striking in a patient recently under my care. If this painful development in the penis is present in the early period of appendicular inflammation, it may aid materially in making a diagnosis at this stage, and I would suggest that surgeons report instances of this kind, which come under observation, so as to throw light upon this matter. Of course this feature cannot avail in the female, but it is found that a vaginal examination affords an insight to the nature of the case when a well defined tumefaction of the appendix exists.

The complications presented in females from ovaritis or distension of the fallopian tube on the right side, introduces an element of doubt in females which is never found in males. I was called to a neurotic lady some months ago, who referred her pain to the site of the appendix and this was accompanied with a marked sensitiveness upon palpation in the right iliac fossa, with slight fullness in this region, which led me to suspect appendicitis. But all passed away with the treatment adopted, and I was greatly relieved as to the knotty question of diagnosis. It has, however, come under my observation in another female patient to verify the diagnosis of appendicitis made by the attending physician, without recognizing the urgency for an operation upon my first visit as consultant. Upon being recalled three days subsequently, the patient was in a state of profound collapse from a supposed perforation of the appendix, and being impressed with the conviction that she would sink under an operation, I declined to use the knife. She was really in a moribund condition and died within two hours afterwards.

This result impressed me with the great importance of taking time by the forelock, even in cases of appendicitis not presenting symptoms of gravity, and having another opportunity soon afterwards of confirming the judgment of a colleague who had diagnosticated appendicitis, an operation was urged.

I have no doubt that some cases will occur in which the patient may recover without any operation, but on the other hand the delay in using the knife must lead to death in many cases which would have been saved by the timely resort to a surgical operation. The great difficulty of deciding in advance which sort of case we are dealing with warrants surgical interference in all cases.

It has recently been a vexed question to decide when an operation is called for in a case supposed to be appendicitis, and it may be laid down as a rule to guide us that upon recognizing the signs of local inflammation with constitutional disturbance, the operation is demanded.

Appendicitis has received so much attention from the profession of late that a successful operation after an established diagnosis in a case of this kind should be recorded. A young man came under the care of Dr. J. S. Todd July 21st, having symptoms indicative of appendicitis, and I was called in consultation by him July 23d and concurred in his diagnosis. It was determined to watch the developments, and on July 24th it was agreed that the progressive local trouble and constitutional disturbance demanded immediate interference. The operation was accordingly made by me with the assistance of Drs. Todd, H. F. Harris, and J. McF. Gaston, Jr. An incision four inches in length, extending perpendicularly downward from the level of the umbilicus on the outer border of the right rectus muscle, exposed the cecum, and the appendix was found by careful manipulation at its most dependent part. It was ligated off from the cecal attachment and excised, revealing a small perforation and containing three distinct fecal concretions or enteroliths. Suppuration had already appeared at the lower portion of the incision, which was removed by sponging, and a thick layer of iodoform gauze was packed into the upper portion of the incision temporarily to prevent any extension of the contamination upwards. The peroxide of hydrogen was used for cleansing the purulent focus, and subsequently free irrigation with hot water was employed for all the adjacent tissues. The space below the cecum was filled with iodoform gauze, extending out at the lower angle, thus securing drainage. The peritoneum was closed in the upper half of the incision with catgut, and the muscles, fascia, and skin with iron-dyed silk suture. The whole was covered with iodoform gauze and cotton, secured by a roller bandage.

The patient was kept under the influence of morphine with atropine for twenty-four hours, after which Epsom salts with senna tea was given by the mouth and by enema, using also the hypodermatic tablets of magnesium sulphate from time to time until the bowels were freely evacuated. There was great discomfort experienced by the patient for several days, but at the end of a week his functions were performed in a normal manner. The stitches were removed one after another from the skin, and adhesive plaster has been used since for bringing the edges of the wound in apposition, keeping gauze in the lower angle.

Upon the tenth day after the operation the ligature was found detached from the stump of the appendix and removed. The patient was allowed for the first time to get upon his feet on the fifteenth day and everything seemed favorable to a speedy recovery on the twenty-second day after the operation. He continued to improve steadily and soon resumed his business, completely restored to his usual health.

It will be noted that there was a departure from what most operators have inculcated in regard to using purgatives previous to an operation for appendicitis and withholding opiates subsequently. The reasoning of my colleague and myself was that complete repose of the intestinal canal for a time was indicated, and we had no reason to regret this mode of treatment.

It strikes me forcibly that surgeons who are doing abdominal work of late, have been led to an extreme in condemning opiates in all the conditions presented in this class of cases. While avoiding the erroneous practice of locking up the bowels by opium in threatened peritonitis and urging free catharsis with salines to prevent peritonitis, it has been overlooked that there is often an element of pain and restlessness which require an anodyne. Allowing that the general principle of treating peritonitis with salines is correct, I am equally convinced that an opiate influence is often attended with a salutary effect in cases of abdominal surgery, and the result was entirely satisfactory in this case. While this report is brief, it serves to illustrate the importance of an early diagnosis and early operation in appendicitis.

INFERENCES.

1. The first inference from a general consideration of ileo-cecal troubles is, that all collections of pus should be evacuated by free incision followed by gauze drainage.

2. Should the appendix be involved in the abscess and already in a necrosed state, it is fair to infer that the canal is closed so that there is no communication with the cecum, and hence excision is not requisite.

3. If, on the contrary, the appendix is found to be enlarged and indurated without perforation, it should be ligated and removed at once.

4. In suspected cases of appendicitis, without the signs of suppuration or the presence of a local swelling or induration, an exploratory operation by a transverse incision above Poupart's ligament, with separation of the muscular fibers, should be resorted to without delay.

5. With a clear diagnosis of appendicitis, a longitudinal incision on the outer border of the right rectus muscle, extending downward over the cecum, is best adapted to reach the appendix.

6. In all cases of recent occurrence, in which suppuration has not appeared, but there exists an inflammatory process of the appendix, it should be removed.

